# How future work self-salience influences occupational sense of mission among medical students in the post-pandemic era: a dual-perspective analysis from variable-centered and person-centered approaches based on professional identity

**DOI:** 10.3389/fpubh.2026.1753631

**Published:** 2026-02-03

**Authors:** Qihe Zhong, Yiwen Zhou, Junxian Li, Yingping Huang

**Affiliations:** School of Medical Imaging, North Sichuan Medical College, Nanchong, China

**Keywords:** COVID-19, future work self-salience, medical students, occupational sense of mission, professional identity

## Abstract

**Background:**

In the post-pandemic era, medical students face increased occupational uncertainty. Future work self-salience as a prospective dimension of self-awareness, shapes career choices and constitutes a core driver of professional engagement. However, few studies have explored the relationship between future work self-salience and occupational sense of mission among medical students or its underlying mechanisms. This study examines the associations among professional identity, future work self-salience, and occupational sense of mission in medical students in the post-pandemic era, as well as heterogeneity in these constructs.

**Methods:**

Using a cross-sectional design and random sampling, 568 medical students were recruited from three comprehensive universities in Sichuan Province, China. Validated instruments measured future work self-salience, occupational sense of mission, and professional identity. A variable-centered approach (PROCESS Model 4) tested the mediating role of professional identity. A person-centered approach employed latent profile analysis to identify subtypes based on future work self-salience and professional identity, with one-way ANOVA examining differences in occupational sense of mission across profiles.

**Results:**

Variable-centered analyses showed that future work self-salience positively predicted occupational sense of mission, with professional identity partially mediating this relationship. Person-centered analyses identified three distinct profiles: (1) High Future Work Self-salience–High Professional Identity, (2) Moderate Future Work Self-salience–Moderate Professional Identity, and (3) Low Future Work Self-salience–Low Professional Identity. ANOVA revealed that the High Future Work Self-salience–High Professional Identity profile exhibited the highest occupational sense of mission.

**Conclusion:**

In the post-pandemic era, medical students’ future work self-salience enhances their occupational sense of mission by strengthening professional identity; however, individual heterogeneity leads to differential effect magnitudes. Educational interventions targeting students with low occupational sense of mission should strengthen future work self-salience and professional identity training to elevate overall occupational sense of mission and support sustainable development of healthcare professionals in the post-pandemic context.

## 1 Introduction

The COVID-19 pandemic has not only exposed the fragility of healthcare workforce systems but has also reshaped the educational and career development landscape for medical students (1, 2), a population that remains comparatively less examined than in-service healthcare professionals in post-pandemic vocational psychology research. Unlike practicing clinicians, medical students are at a formative stage of professional identity construction and career meaning-making; thus, understanding the psychological drivers of their sustained professional engagement is both theoretically important (3, 4). On one hand, societal expectations of the medical profession have reached unprecedented levels, with medicine being re-imbued with symbols of social responsibility, public mission, and humanitarian care (5). Specifically, as aspiring healthcare professionals and college students, their public spirit—manifested in a proactive commitment to public health welfare, sense of collective responsibility, and dedication to addressing societal health needs—has become an indispensable component of meeting these elevated societal expectations for the medical profession. On the other hand, in the post-pandemic era, medical students face a distinctive constellation of career-related stressors arising from overburdened healthcare systems and increasingly complex occupational ecologies (6, 7). Specifically, strained clinical resources and disrupted training opportunities may limit authentic workplace exposure, elevated perceived occupational risks may amplify uncertainty about future roles, and the normalization of online or hybrid learning may weaken professional socialization and role internalization (8–10). For example, resource shortages and disrupted clinical rotations have limited hands-on experience, while heightened awareness of occupational hazards may further undermine confidence in future roles (11). This dual impact has compelled medical students in the post-pandemic era to re-evaluate their professional positioning and value pursuits. Especially when career uncertainty, hybridized learning modes, and employment pressures converge (12, 13), how individuals reconstruct their professional self and sense of social contribution within this new sociocultural context has become a critical question in educational psychology. In China, medical training typically includes a pre-clinical stage followed by progressively intensified clinical placements (e.g., clerkships and internships) in teaching hospitals (14, 15). During the internship period, students may participate in ward-based routines that resemble clinical work patterns (e.g., early starts, rotating schedules, and high task demands), and workload can vary substantially across specialties and institutions. This training context is important because high clinical workload may elicit work-related distress and burnout-like experiences, which could influence how students construct their future work selves, internalize professional identity, and perceive a sense of occupational mission. Accordingly, the present study investigates how future work self-salience influences occupational sense of mission among medical students in the post-pandemic era, aiming to elucidate psychological mechanisms in medical education and promote the integration of theory and practice for sustainable career development.

Future work self-salience is a key prospective component of the self-concept system (16), referring to the degree to which an individual’s hoped-for future work self is cognitively accessible, personally important, and readily applied to guide current behavior and decision-making (17, 18). Greater salience of the future work self enables individuals to more effectively bridge present actions with long-term professional aspirations in dynamic career environments (19). Empirical evidence indicates that future work self-salience significantly predicts career motivation, proactive career behaviors, and persistence in career planning (20, 21). For medical students, whose profession is characterized by strong ethical norms and societal expectations, professional value derives not only from personal achievement but also from internalized social responsibility and altruistic orientation (22, 23). Consequently, when the future work self is more salient, medical students are more likely to generate intrinsic professional drive, manifesting a stronger occupational sense of mission in learning and practice (24). Occupational sense of mission, as a core psychological resource that sustains persistent effort and adherence to professional beliefs (25), is a vital predictor of psychological well-being, learning motivation, and career commitment among medical students (26, 27).

Grounded in career construction theory, career development unfolds through anticipation, planning, and meaning-making regarding one’s future work self (28, 29). Future work self-salience reflects the accessibility and motivational potency of future career goals and their coherence with current behavior (30, 31). High salience guides greater goal focus and responsibility while maintaining stability of professional meaning amid external change (32). For medical students, a highly salient “future doctor self” serves not only as psychological fuel for sustained academic investment but also as a cognitive foundation for comprehending the medical mission and internalizing professional values (33, 34). When students can readily access and apply vivid images of their ideal future roles, they more easily activate altruistic intentions, awareness of social contribution, and respect for human dignity—core elements that collectively constitute occupational sense of mission (35).

Professional identity refers to the integrative cognitive and affective experience of one’s occupational role, values, and responsibilities (36, 37). According to social identity theory, professional identity emerges from internalization and emotional attachment to one’s occupational role within group belonging and social interaction (38, 39). For medical students, professional identity extends beyond mastery of knowledge and skills to deep understanding of and emotional commitment to “becoming a doctor” (40). Higher professional identity enables greater perceived congruence between personal actions and societal values, thereby eliciting a stronger occupational sense of mission (41). Moreover, professional identity may serve as a psychological bridge linking future work self-salience and occupational sense of mission: a highly salient future work self enhances goal orientation and self-concept coherence, facilitating role internalization and identity formation, which in turn strengthens the depth and endurance of occupational sense of mission (42, 43).

Previous studies have predominantly adopted variable-centered approaches, focusing on linear relationships and overall trends (44, 45). However, medical students exhibit considerable psychological heterogeneity. Relying solely on variable-centered methods cannot reveal latent configurations of future work self-salience and professional identity. Therefore, this study introduces a person-centered perspective using latent profile analysis (LPA) to identify subtypes and examine differential patterns of the future work self-salience–occupational sense of mission relationship across profiles. The integration of both perspectives validates macro-level model robustness while uncovering micro-level differentiated pathways, providing a precise foundation for tailored interventions in medical education.

**Figure 1:**
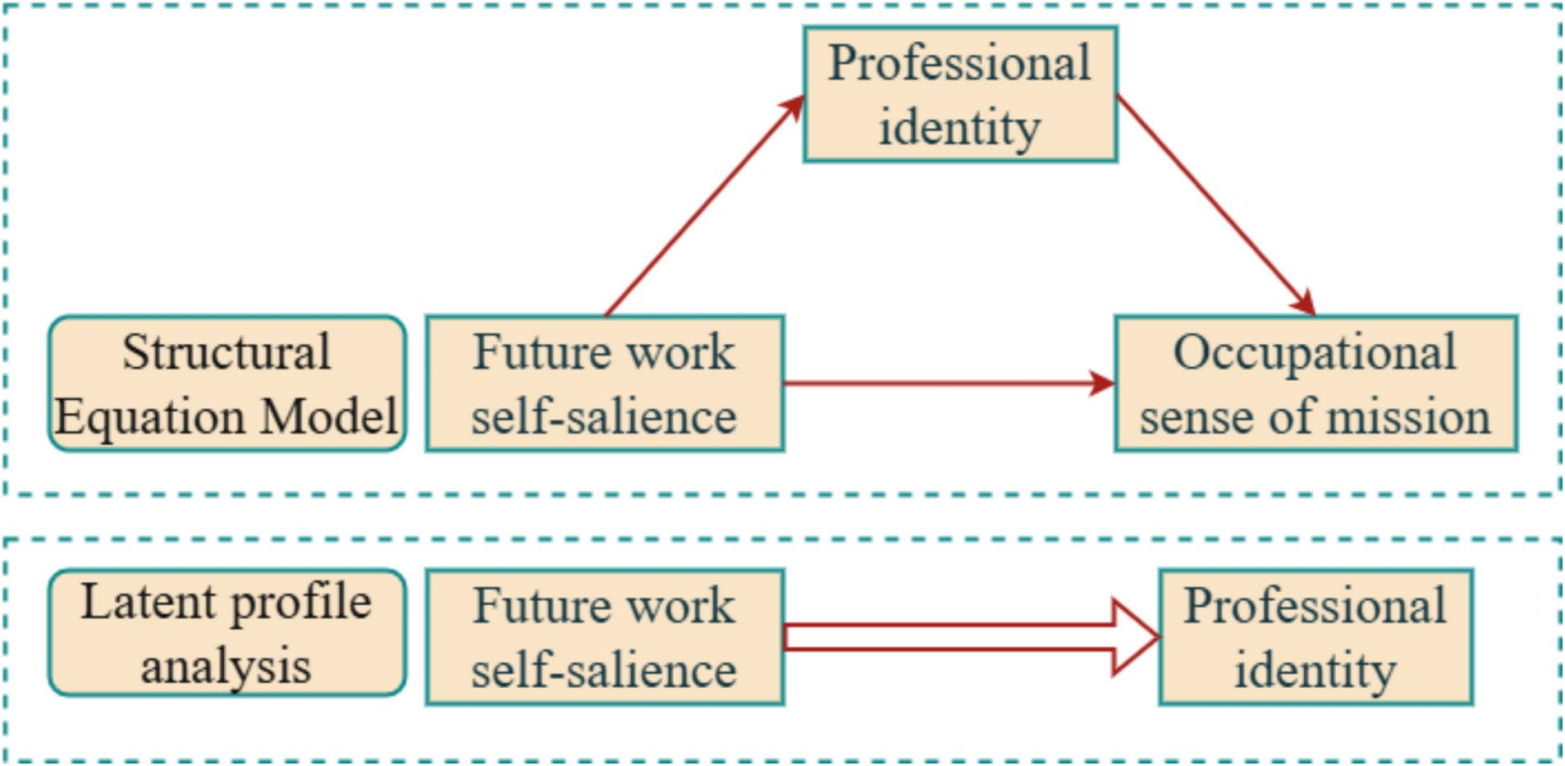
Study framework model.

The framework model of this study is shown in Figure 1.

Based on the above, the following hypotheses are proposed:

*H1*: Medical students’ future work self-salience significantly and positively predicts their occupational sense of mission.

*H2*: Professional identity significantly mediates the relationship between future work self-salience and occupational sense of mission.

*H3*: Medical students exhibit significant heterogeneity in future work self-salience and professional identity.

## 2 Methods

### 2.1 Study design

This study adopted a cross-sectional quantitative design that integrated variable-centered and person-centered analytical approaches to examine the mechanisms linking future work self-salience, professional identity, and occupational sense of mission among medical students in the post-pandemic era. A mediated model was first tested using structural equation modeling to evaluate model fit and path coefficients. The LPA was then employed to identify heterogeneous subtypes based on future work self-salience and professional identity, followed by comparisons of occupational sense of mission across subtypes. The study adhered to established empirical paradigms in vocational psychology and utilized anonymous self-report questionnaires to minimize social desirability bias.

### 2.2 Ethical considerations

This study strictly adhered to the ethical principles of the Declaration of Helsinki and received approval from the Ethics Committee of North Sichuan Medical College. Prior to the survey, participants were informed of the study purpose, anonymity, data confidentiality, and their right to withdraw. Participants completed the questionnaire voluntarily and provided informed consent online. All data were used solely for scientific research and were not related to academic evaluation or administrative decisions. Personal information was anonymized and coded during data processing to ensure privacy protection.

### 2.3 Participants and sampling procedure

#### 2.3.1 Recruitment

Data were collected between August and September 2025 from three comprehensive medical universities in Southwest China. A combination of convenience and stratified random sampling was employed to enhance sample diversity and representativeness. After obtaining institutional approval, electronic questionnaire links were distributed via WeChat with the assistance of faculty and academic advisors. To reduce common method bias, items from different scales were randomized, and several reverse-scored items were included.

#### 2.3.2 Inclusion and exclusion criteria

To ensure accuracy in participant selection, strict inclusion criteria were applied: (1) Medical students aged ≥18 years; (2) Currently enrolled as full-time medical students; (3) At least three months of clinical internship experience; (4) Clear consciousness and no cognitive or communication impairments.

Exclusion criteria were as follows: (1) Previous diagnosis of severe psychiatric disorders (e.g., clinical anxiety or depression) that might introduce bias; (2) Direct involvement in frontline healthcare for more than one year during the COVID-19 pandemic (to avoid extreme stress effects); and (3) Highly patterned response tendencies or excessively short questionnaire completion time (< 3 min).

#### 2.3.3 Sample size justification

The target sample size was determined to be adequate for the planned variable-centered mediation analysis and the person-centered LPA. For mediation models estimated with ordinary least-squares regression and bootstrapping, we evaluated them with G*power 3.1, with an effect size of 0.15 (f^2^), an *α* of 0.05, a power value of 0.95 (1 − *β*), and a minimum sample size of 172. For LPA, simulation and applied guidelines also suggest that samples in the range of N ≈ 300–500 + typically allow reliable recovery of a small number of profiles with acceptable classification accuracy when indicators are continuous and profile separation is not trivial (46). Given these considerations and the intent to compare multiple profiles while maintaining sufficient subgroup sizes, we aimed to recruit at least 500 participants. The final analytic sample (*N* = 568) therefore provided an empirically appropriate basis for the mediation and LPA results reported in this study.

#### 2.3.4 Sample

A total of 597 participants were recruited. Based on the inclusion and exclusion criteria, 13 participants who had engaged in medical work for more than 1 year during the pandemic, 12 participants with highly consistent or overly rapid responses, and 4 participants with incomplete questionnaires were excluded. The final valid sample consisted of 568 participants, yielding a valid response rate of 95.14%.

A total of 568 medical students were included in this study. The sample was predominantly female (70.6%), and participants from urban areas slightly outnumbered those from rural areas (57.9% vs. 42.1%). Regarding COVID-19–related experiences, approximately two thirds of students reported having experienced COVID-19–related events (69.2%), and most reported having been infected with COVID-19 (74.6%). Notably, recent exposure to COVID-19–related information was heterogeneous, with 46.8% reporting exposure and 53.2% reporting no exposure. Detailed demographic information is provided in Table 1.

**Table 1:** Demographic information for all participants.

Variables	Items	Frequency	Percentage
Gender	Male	167	29.40%
	Female	401	70.60%
Place of residence	Urban	329	57.90%
	Rural area	239	42.10%
Internship experience	Yes.	392	69.00%
	No.	176	31.00%
Monthly income level	≤5,000¥	226	39.80%
	5,001–8,000¥	172	30.30%
	8,001–12,000¥	108	19.00%
	12,001 and above	62	10.90%
Have experienced COVID-19	Yes.	393	69.20%
	No.	175	30.80%
Whether you have COVID-19	Yes.	424	74.60%
	No.	144	25.40%
Whether you have recently received information about COVID-19	Yes.	266	46.80%
	No.	302	53.20%
Age	19.56 ± 1.601		

In this study, “recently received information about COVID-19” captured whether participants reported recent exposure to COVID-related information (Yes/No), rather than general awareness. The observed proportion (46.8%, 266/568) should therefore be interpreted as a time-bound indicator of current information salience and media/educational exposure at the time of data collection, not as a population-level estimate of COVID knowledge. Direct comparisons with non-medical populations should be made cautiously because medical students may differ systematically in information channels and in perceived relevance of such information. We consequently treat these COVID-related indicators primarily as descriptive context variables and, where applicable, as exploratory covariates in supplementary analyses.

### 2.4 Measures tools

#### 2.4.1 Future work self-salience scale

Future work self-salience was assessed using the five-item unidimensional scale developed by Strauss, Griffin (31), which evaluates individuals’ clarity regarding their future professional roles. A sample item is: “I can clearly imagine what I will be like in my future medical career.” The Chinese version has been validated for cultural adaptation and psychometric reliability among Chinese students (47). Responses were rated on a five-point Likert scale (1 = strongly disagree, 5 = strongly agree), with total scores ranging from 5 to 25; higher scores indicate greater clarity of future work self. In the present study, Cronbach’s *α* was 0.851, indicating good internal consistency. Confirmatory factor analysis (CFA) demonstrated good model fit (χ^2^/df = 3.139, CFI = 0.993, GFI = 0.991, AGFI = 0.967, RMSEA = 0.061).

#### 2.4.2 Occupational sense of mission scale

Occupational sense of mission was measured using the 29-item Chinese Occupational Sense of Mission Scale developed by Zhang (48). A sample item is: “I look forward to my future professional life.” The scale has been widely applied in Chinese populations (49), including in studies of nurses’ occupational sense of mission (27). Items were rated on a five-point Likert scale (1 = strongly disagree, 5 = strongly agree). Total scores range from 29 to 145, with higher scores indicating stronger occupational sense of mission. In this study, Cronbach’s *α* was 0.897. CFA results showed good model fit (χ^2^/df = 1.555, CFI = 0.981, GFI = 0.948, AGFI = 0.938, RMSEA = 0.043).

#### 2.4.3 Professional identity scale

Professional identity was assessed using the 18-item Chinese Professional Identity Scale for health professionals developed by Liao and Wang (50), comprising four dimensions: Professional Commitment and Devotion, Emotional Identification and Belongingness, Professional Goals and Values, and Self-fulfillment and Retention Tendency. A sample item is: “Even after marriage, I will continue to work in the healthcare field.” This scale has been widely used among health professional students (51) and undergraduate nursing students (52). Items were rated on a five-point Likert scale (1 = strongly disagree, 5 = strongly agree), with total scores ranging from 18 to 90; higher scores indicate stronger professional identity. In this study, Cronbach’s *α* was 0.945. CFA results indicated acceptable model fit (χ^2^/df = 5.216, CFI = 0.923, GFI = 0.880, AGFI = 0.831, RMSEA = 0.086).

#### 2.4.4 Cultural adaptation and measurement validity in the present sample

All instruments used in this study were administered in Chinese. For each construct, we adopted existing Chinese versions that have been previously translated, culturally adapted, and psychometrically validated in Chinese student populations. To further ensure appropriateness for the current context (medical undergraduates/interns in Southwest China in the post-pandemic era), the research team conducted an additional content-adequacy check prior to data collection. Specifically, two faculty members with expertise in medical education and educational psychology reviewed item wording for semantic clarity, contextual relevance to medical training, and potential ambiguity. The online questionnaire platform was configured to randomize the presentation order of items from different scales and to include reverse-scored items as prespecified by the original instruments to reduce acquiescence and patterned responding.

In the analytic phase, we re-examined internal consistency and factorial validity in the present sample. Cronbach’s *α* values indicated good to excellent reliability for future work self-salience (α = 0.851), occupational sense of mission (α = 0.897), and professional identity (α = 0.945). Confirmatory factor analyses (CFA) supported the intended measurement structure for each scale with acceptable-to-good fit indices. These results provide empirical support that the Chinese instruments functioned adequately in our sample and are suitable for subsequent mediation and latent profile analyses.

### 2.5 Statistical analysis

Data were analyzed using SPSS 27.0, AMOS 29.0, and Mplus 8.3. SPSS 27.0 was used for data cleaning, descriptive statistics, and normality testing, as well as the Harman single-factor test to assess common method bias. AMOS 29.0 was used to conduct confirmatory factor analyses to evaluate the fit of the measurement models, using CFI, TLI, RMSEA, and GFI as fit indices. The direct effect of future work self-salience on occupational sense of mission and the mediating role of professional identity were examined using PROCESS Model 4; bootstrapping with 5,000 resamples was used to calculate 95% confidence intervals for indirect effects. Latent profile analysis was performed using Mplus 8.3 to identify latent subgroups based on future work self-salience and professional identity. Finally, one-way ANOVA was conducted to examine differences in future work self-salience and professional identity across latent subgroups. All statistical tests were two-tailed, with a significance threshold of *p* < 0.05.

## 3 Results

### 3.1 Common method bias analysis

To assess the risk of common method bias associated with single-source, self-report data, Harman’s single-factor test was conducted by entering all scale items into an exploratory factor analysis with unrotated principal component extraction. Seven factors with eigenvalues greater than 1 emerged. The first factor explained 37.360% of the total variance, which is below the commonly used heuristic threshold (≈40%). This pattern suggests that a single general factor did not dominate the covariance among items and that severe common method bias is unlikely to fully account for the observed associations.

At the same time, we acknowledge that Harman’s test is a coarse diagnostic and cannot rule out more subtle forms of method variance. Therefore, in addition to this statistical check, we implemented procedural remedies (anonymous participation, confidentiality assurance, randomized item ordering across scales, inclusion of reverse-scored items, and screening for overly rapid/patterned responding) to reduce the likelihood of common method bias.

### 3.2 Descriptive statistics and correlation analysis

Descriptive statistics and correlations for future work self-salience, professional identity, and occupational sense of mission are presented in Table 2. The mean score for future work self-salience was 3.275 (SD = 0.659), for professional identity was 3.717 (SD = 0.567), and for occupational sense of mission was 3.535 (SD = 0.427). The mean scores for all core study variables were above the midpoint of 2.5, indicating that medical students reported relatively high levels of future work self-salience, professional identity, and occupational sense of mission.

**Table 2:** Descriptive statistics and correlations for core variables.

Variables	M	SD	Skewness	Kurtosis	1	2	3
Future work self-salience	3.275	0.659	−0.035	0.646	–		
Professional identity	3.717	0.567	0.171	0.087	0.520***	–	
Occupational sense of mission	3.535	0.427	0.059	1.669	0.425***	0.580***	–

***p < 0.001.

Regarding the criteria for approximate normality proposed by Kline (53) (skewness < |3|, kurtosis < |8|), the skewness of the study variables ranged from −0.035 to 0.171, and kurtosis ranged from 0.087 to 1.669, indicating that the data followed an approximately normal distribution.

Correlation analysis revealed that future work self-salience was strongly and positively correlated with professional identity (*r* = 0.520, *p* < 0.001), indicating enhanced emotional engagement among students. Future work self-salience also showed a moderate positive correlation with occupational sense of mission (*r* = 0.425, *p* < 0.001), reflecting stronger goal orientation and meaning-seeking tendencies. Professional identity had a strong positive correlation with occupational sense of mission (*r* = 0.580, *p* < 0.001), suggesting increased organizational empowerment and meaning amplification.

All correlation coefficients were below |0.8|, supporting subsequent hypothesis testing and indicating an absence of multicollinearity concerns (VIF < 5).

### 3.3 The mediating role of professional identity

A one-way ANOVA indicated no significant differences in core study variables based on demographic information (*p* > 0.05). To examine the mediating role of professional identity, we used Process Model 4 (54), with future work self-salience as the independent variable, occupational sense of mission as the dependent variable, and Professional Identity as the mediator.

**Figure 2:**
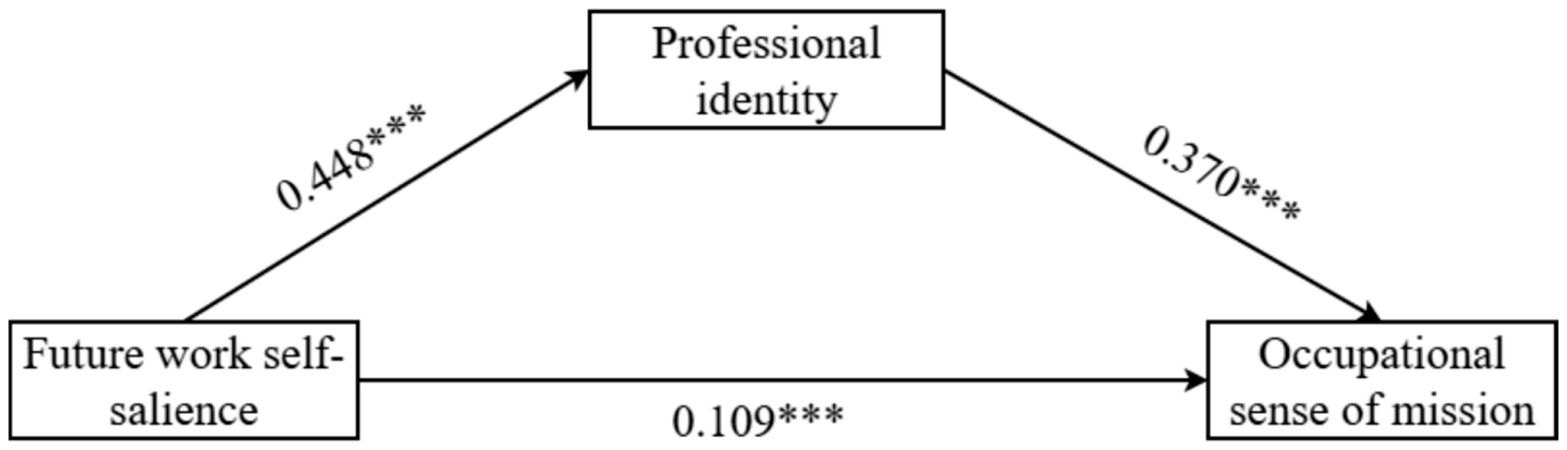
Path coefficient plot for the mediation model of professional identity, ***p < 0.001.

The results indicated that future work self-salience significantly and positively predicted professional identity (*β* = 0.448, *p* < 0.001, 95% CI = [0.387, 0.508]). Future work self-salience also significantly and positively predicted occupational sense of mission (*β* = 0.109, *p* < 0.001, 95% CI = [0.059, 0.159]). Furthermore, professional identity significantly and positively predicted occupational sense of mission (*β* = 0.370, *p* < 0.001, 95% CI = [0.312, 0.429]). The path coefficients for the mediating effect of professional identity are presented in Table 3 and Figure 2.

**Table 3:** Regression coefficients for the professional identity mediation model.

Model	Outcome	Predictor	Overall fit			Path coefficients		
			R	R*^2^*	F	β	t	95% CI
1	Professional identity	Future work self-salience	0.520	0.270	209.737***	0.448	14.482***	[0.387, 0.508]
2	Occupational sense of mission	Future work self-salience	0.598	0.357	156.859***	0.109	4.273***	[0.059,0.159]
		Professional identity				0.370	12.461***	[0.312, 0.429]

***p < 0.001.

The mediating effect of professional identity was tested using bootstrapping with 5,000 resamples. The total effect of future work self-salience on occupational sense of mission was 0.275. Among this, 60.3% was explained by the mediating role of professional identity. Specifically, Future work self-salience had a significant direct effect on occupational sense of mission (*β* = 0.109, SE = 0.026, 95% CI = [0.059, 0.160]), accounting for 39.7% of the total effect. The path future work self-salience → professional identity → occupational sense of mission showed a significant partial mediating effect (*β* = 0.166, SE = 0.030, 95% CI = [0.109, 0.226]). The decomposition of mediating effects is shown in Table 4.

**Table 4:** Effect decomposition of the mediating effect of professional identification.

Effect decomposition	β	SE	LLCI	ULCI	Effect proportion
Total effect	0.275	0.025	0.227	0.324	100.0%
Direct effect	0.109	0.026	0.059	0.160	39.7%
Indirect effect	0.166	0.030	0.109	0.226	60.3%

### 3.4 Latent profile analysis of future work self-salience and professional identity

Latent Profile Analysis was conducted to identify potential heterogeneity in Future Work Self-salience and Professional Identity. Using Mplus 8.3, models with 1 to 5 latent profiles were estimated. The optimal number of profiles was determined based on the Bayesian Information Criterion (BIC), Akaike Information Criterion (AIC), sample-size adjusted BIC (aBIC), entropy (entropy > 0.80), and the Bootstrap Likelihood Ratio Test (BLRT) to verify differences between profiles. The model fit indices for the 1- to 5-profile models are presented in Table 5.

**Table 5:** Model fit indices for latent profile analysis of future work self-salience and professional identity.

Profile	AIC	BIC	aBIC	Entropy	LMR (p)	BLRT (p)	Proportion of potential subgroups
1	31209.121	31408.859	31262.830	–	–	–	–
2	27207.994	27511.943	27289.725	0.956	<0.001	<0.001	46.2%/53.8%
3	**25701.872**	**26110.032**	**25811.625**	**0.957**	**<0.001**	**<0.001**	**38.5%/50.3%/11.2%**
4	25130.579	25642.949	25268.354	0.945	0.058	<0.001	21.9%/22.5%/44.8%/10.8%
5	24652.572	25269.154	24818.369	0.951	0.579	<0.001	1.1%/21.5%/22.2%/44.4%/10.8%

AIC = Akaike information criterion; BIC = Bayesian information criterion; aBIC = sample-size adjusted BIC; LMR = Lo–Mendell–Rubin likelihood ratio test; BLRT = bootstrap likelihood ratio test. The 3-profile model (in bold) was selected as the optimal solution.

The 1-profile model showed higher AIC, BIC, and aBIC values (AIC = 3120.9121, BIC = 31408.859, aBIC = 3126.2830), indicating poor fit. When increasing to a 2-profile model, these values decreased significantly (AIC = 2720.7994, BIC = 27511.943, aBIC = 2728.9725), and both the LMR and BLRT *p*-values were less than 0.001, suggesting improved heterogeneity. Progressing to a 3-profile model, the information criteria continued to decrease (AIC = 2570.1872, BIC = 26110.032, aBIC = 2581.1625), the LMR and BLRT remained significant (*p* < 0.001), entropy reached a peak of 0.957, and the class proportions were balanced (38.5, 50.3, and 11.2%), with the smallest class exceeding 10%, thus avoiding an overly small subgroup issue. However, from the 3-profile model to the 4- and 5-profile models, although some indices improved slightly, the LMR *p*-values were no longer significant (0.058 and 0.579, respectively), the BIC began to increase, entropy slightly decreased, and the 5-profile model contained a very small subgroup (1.1%), suggesting potential overfitting and noise capture. Consequently, the 3-profile model was selected as the best-fitting model.

**Figure 3:**
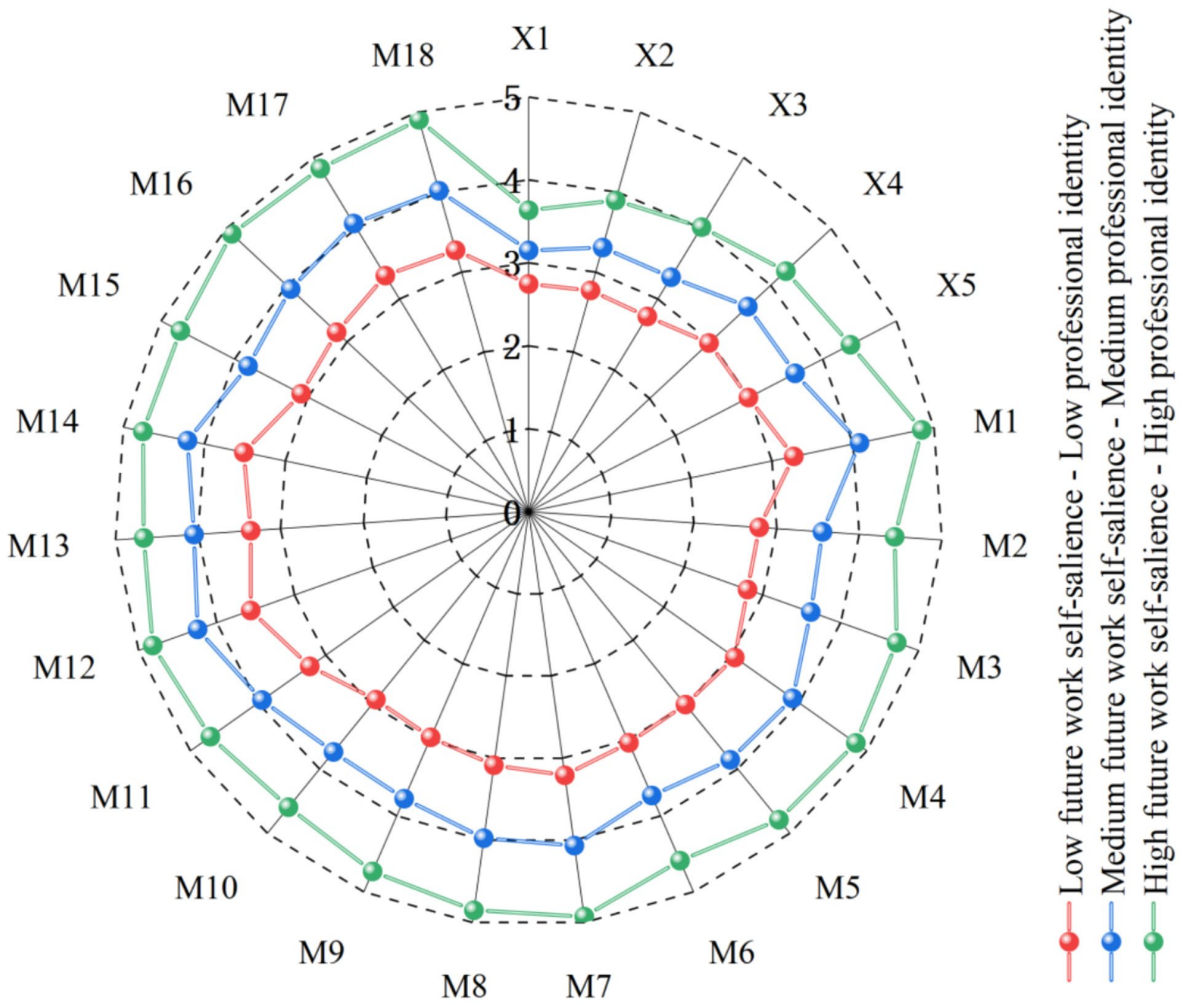
Profile plot of the three latent subgroups identified in the 3-profile latent profile model.

To enhance the clarity of the LPA results, the 3-profile model was visualized using Origin 2021 software, as shown in Figure 3. The first latent profile was labeled “High Future Work Self-salience–High Professional Identity,” accounting for 11.2% of the total sample. The second profile was labeled “Moderate Future Work Self-salience–Moderate Professional Identity,” comprising 50.3% of the sample. The third profile was labeled “Low Future Work Self-salience–Low Professional Identity,” representing 38.5% of the sample.

### 3.5 One-way ANOVA of latent subgroups and occupational sense of mission

A one-way ANOVA was conducted with the three latent subgroups as the independent variable and occupational sense of mission as the dependent variable. The results showed that compared to the “Low Future Work Self-salience–Low Professional Identity” subgroup (M = 3.534, SD = 0.637), the “Moderate Future Work Self-salience–Moderate Professional Identity” subgroup reported a relatively higher Occupational Sense of Mission (M = 3.709, SD = 0.563), while the “High Future Work Self-salience–High Professional Identity” subgroup reported the highest level (M = 3.764, SD = 0.547). Therefore, there was a significant difference in occupational sense of mission among the three latent subgroups (*F* (2, 565) = 4.323, *p* = 0.014).

## 4 Discussion

The results indicated that future work self-salience significantly and positively predicted occupational sense of mission, and professional identity partially mediated this association (the indirect effect accounted for 60.3% of the total effect). Latent profile analysis identified three student profiles: High Future Work Self-salience–High Professional Identity (11.2%), Moderate Future Work Self-salience–Moderate Professional Identity (50.3%), and Low Future Work Self-salience–Low Professional Identity (38.5%). One-way ANOVA showed significant differences in occupational sense of mission across profiles; the High Future Work Self-salience–High Professional Identity profile reported the highest level, demonstrating a graded pattern from the Low Future Work Self-salience–Low Professional Identity to the High Future Work Self-salience–High Professional Identity profile. These findings suggest that profile-based, tiered interventions may be warranted, prioritizing the enhancement of future work self-salience and professional identity among students in the Low Future Work Self-salience–Low Professional Identity and Moderate Future Work Self-salience–Moderate Professional Identity profiles to promote occupational sense of mission and sustained professional engagement.

### 4.1 Person-centered analysis

The latent profile analysis revealed substantial psychological heterogeneity among medical students regarding future work self-clarity and professional identity. Three distinct latent subgroups were identified: High Future Work Self-salience–High Professional Identity, Moderate Future Work Self-salience–Moderate Professional Identity, and Low Future Work Self-salience–Low Professional Identity. From a person-centered perspective, these findings transcend the limitations of traditional variable-centered approaches that focus solely on average effects, and instead highlight structural differences and potential mechanisms underlying medical students’ professional psychological development.

The emergence of three distinct profiles in our study resonates with prior person-centered research on professional identity and career development, yet also reveals unique patterns attributable to the post-pandemic context. Morin, Meyer (55) identified similar tripartite configurations when examining identity profiles among healthcare workers, distinguishing high-commitment, ambivalent, and disengaged subgroups. Our findings extend this framework by demonstrating that future work self-salience and professional identity co-vary systematically, forming coherent psychological configurations rather than independent dimensions. This pattern aligns with theoretical propositions from career construction theory, which posits that future-oriented cognition and identity formation are developmentally intertwined processes.

Notably, the proportion of students in each profile differs from patterns observed in pre-pandemic studies. Liu, Zhang (52) reported that approximately 45% of nursing undergraduates exhibited high sense of coherence and strong professional identity, whereas our study found only 11.2% of medical students in the High Future Work Self-salience–High Professional Identity group. This discrepancy may reflect the lingering psychological impact of the COVID-19 pandemic on medical students’ career certainty and professional commitment (56, 57). The pandemic disrupted traditional pathways of professional socialization, including clinical rotations and mentorship experiences, potentially impeding the consolidation of professional identity for a substantial proportion of students (10). In contrast, Fernet, Guay (58) found that among teachers—another helping profession—approximately 35% demonstrated high occupational commitment profiles post-crisis, suggesting that the magnitude of identity disruption may vary across professional contexts and warrants further cross-disciplinary investigation.

Students in the High Future Work Self-salience–High Professional Identity group demonstrated consistent alignment between their career ideals and their understanding of professional reality. Students in the High Future Work Self-salience–High Professional Identity profile showed a coherent alignment between their future-oriented career representations and their internalization of professional values, and they reported the highest occupational sense of mission. Conceptually, this configuration can be interpreted as a “meaning-coherent” vocational self-structure in which a vivid future doctor self is supported by strong role identification and value congruence. Importantly, because the present study is cross-sectional, we refrain from attributing this profile pattern to a causal “post-COVID effect.” Nevertheless, the post-pandemic sociocultural context may provide a plausible backdrop for such meaning coherence to emerge. After major societal crises, some groups may display enhanced solidarity, value reaffirmation, or meaning-making processes, which can in turn strengthen professional commitment and perceived mission. In medical education, heightened public attention to healthcare work and intensified ethical salience during and after the pandemic may have served as contextual cues that amplify the accessibility and motivational force of a future doctor self. This interpretation remains speculative and should be tested using longitudinal or cohort-comparative designs. This finding corroborates Strauss and Kelly (59) seminal work demonstrating that salience of future work selves predicts proactive career behaviors through enhanced goal clarity and self-regulatory capacity. These students typically exhibit high goal orientation, internal locus of control, and self-determination, enabling them to actively transform vocational aspirations into stable professional identity through learning and clinical practice. Furthermore, this profile mirrors the “engaged professional” subtype identified by Holman, Johnson (60) in their study of healthcare trainees, characterized by high intrinsic motivation and strong value internalization.

In contrast, students in the Low Future Work Self-salience–Low Professional Identity group displayed ambiguity and instability in constructing their professional self-concept. They lacked clear future planning and possessed weak professional belongingness, resulting in a limited understanding of the meaning of the medical profession. This pattern echoes findings from Burford (61), who observed that medical students with underdeveloped professional identity often experienced “identity dissonance”—a mismatch between their idealized physician role and perceived personal capabilities. Such students were more vulnerable to external influences and emotional fluctuations, leading to low levels of professional engagement and calling. The relatively large proportion of students (38.5%) in this low-salience, low-identity profile is particularly concerning and exceeds rates reported in pre-pandemic studies. For instance, Park and Hong (39) found that approximately 25% of medical students reported identity uncertainty, suggesting that pandemic-related disruptions may have exacerbated developmental challenges for a significant subgroup.

The Moderate Future Work Self-salience–Moderate Professional Identity group represented a transitional segment characterized by psychological malleability. Although they had formed an initial framework of professional self-understanding, their identity had yet to achieve deeper consolidation. This “emergent identity” profile corresponds to developmental stages described in Kegan (62) constructive-developmental theory, wherein individuals are in the process of transitioning from externally defined to internally authored professional selves. This finding suggests that this subgroup constitutes a critical target for educational interventions, as structured career guidance, emotional support, and role-model interactions may facilitate their progression toward the High Future Work Self-salience–High Professional Identity profile. Consistent with this interpretation, Cruess, Cruess (34) emphasized that professional identity formation is not linear but occurs through iterative cycles of experience, reflection, and integration—a process that can be deliberately supported through educational design.

Importantly, our three-profile solution demonstrates stronger discriminant validity compared to two-profile models reported in some prior studies. While certain researchers have dichotomized professional identity into high versus low categories (63), our analysis reveals a meaningful intermediate group that would be obscured by binary classification. This nuanced understanding has significant implications for tailored interventions, as students in transitional states may require qualitatively different support strategies than those at developmental extremes (64).

Overall, the person-centered analysis not only confirmed the layered nature of vocational psychological structures but also validated the principle of heterogeneity in the career development of medical students. Professional psychological growth is not linear or homogeneous; instead, it unfolds through multiple developmental pathways grounded in distinct combinations of psychological structures. These insights provide empirical evidence for implementing precision-based educational interventions, encouraging educators to tailor training strategies based on students’ psychological profiles and promoting the dynamic co-evolution of professional identity and occupational sense of mission.

### 4.2 Variable-centered analysis

The variable-centered results demonstrated that future work self-salience significantly and positively predicted occupational sense of mission, with professional identity functioning as a partial mediator. This indicates that future work self-salience contributes to the formation of occupational sense of mission both directly and indirectly through strengthened professional identity. The structural relationships observed in this study validate the combined mechanisms of career construction theory and social identity theory. Future work self represents the psychological starting point of vocational development, whereas professional identity serves as the affective bridge through which future-oriented cognition is transformed into personal meaning.

Future work self-salience reflects students’ ability to articulate clear career goals and visualize future professional roles. Such clarity enhances goal-directed behaviors and internal psychological coherence, enabling medical students to construct meaning-driven logic in academic and career exploration (65). When students have a concrete vision of their future selves, they are better able to regulate learning behaviors and simultaneously cultivate emotional attachment and value alignment with the medical profession (66).

Professional identity plays a key mediating role by internalizing these career values. It bridges rational career planning with emotional commitment, thereby integrating the moral responsibilities inherent in the medical profession with students’ personal aspirations. Students with high professional identity are more likely to internalize career goals as core beliefs (67), exhibit prosocial orientation and social responsibility, and develop a stable and enduring occupational sense of mission.

These findings extend the theoretical boundaries of future work self research by situating it within the context of medical education, thereby illuminating the mediating mechanism of identity and meaning-making. The results underscore that helping students “see their future” is insufficient to cultivate occupational sense of mission; educators must concurrently foster emotional identification to achieve reciprocal integration of cognitive goals and affective values. Future studies should examine the stability of this model across different medical disciplines and at various stages of professional development.

### 4.3 Practical and clinical implications

The findings hold significant implications for medical education practice and vocational psychological counseling. Importantly, our results both corroborate and extend previous intervention research, providing an evidence-based foundation for targeted educational strategies in the post-pandemic era. First, the normalization of infectious-disease risk management and intermittent waves of COVID-related news may have altered how frequently students encounter health information, which can in turn shape the salience of professional values and future-oriented career representations. Second, the post-pandemic period has been characterized by ongoing reforms in clinical training arrangements (e.g., hybrid learning, disrupted rotations, and renewed emphasis on public health preparedness), which may differentially benefit students who already possess clearer future work selves and stronger professional identity. These contextual factors suggest that individual differences in future work self-salience and professional identity are embedded within changing educational and societal conditions, and this reinforces the need for targeted, profile-informed interventions rather than one-size-fits-all programs.

From an educational psychology perspective, future work self-salience serves not only as a cognitive foundation for academic motivation and learning engagement but also as a critical starting point for cultivating occupational sense of mission. This finding aligns with Oyserman, Destin (68) extensive research on future self-continuity, which demonstrates that interventions enhancing the vividness and accessibility of future selves significantly improve goal-directed behavior and academic persistence. Medical schools should therefore implement systematic career development programs to help students clarify professional goals and future orientations. For example, structured career development courses and reflective activities based on future self-narratives can facilitate students’ visualization of future professional roles. Specifically, “possible selves” interventions—wherein students are guided to articulate, visualize, and behaviorally connect with their hoped-for professional selves—have demonstrated efficacy in educational contexts (69) and warrant adaptation for medical education.

Our findings regarding the mediating role of professional identity suggest that future-oriented interventions alone may be insufficient; concurrent attention to identity formation is essential. Mentorship programs should incorporate psychological capital development, allowing students to internalize medical values through interactions with clinical role models and reinforce their emerging sense of “the kind of doctor I want to become.” This recommendation resonates with Cruess, Cruess (34) comprehensive framework for professional identity formation in medical education, which emphasizes the roles of experiential learning, reflection, and mentorship in identity development. Furthermore, Wald, Anthony (70) demonstrated that reflective writing interventions focused on professional identity enhanced medical students’ sense of meaning and commitment—an approach that could be systematically integrated into curricula to strengthen the future work self–professional identity–occupational mission pathway identified in our study.

The present findings illuminate the psychological pathways through which future work self-salience cultivates occupational calling among nursing students. When students can readily access and apply vivid images of their ideal future roles, they engage in a form of prospective self-reflection that activates value-laden professional orientations. Specifically, the clarity and accessibility of future work selves enable students to mentally “try on” the caring, altruistic, and dignity-affirming aspects of nursing practice before fully entering the profession. This anticipatory identification, mediated by strengthened professional identity, transforms abstract professional values into personally meaningful commitments, ultimately manifesting as a robust sense of occupational mission characterized by altruistic intentions, awareness of social contribution, and deep respect for human dignity. Moreover, strengthening professional identity should be a central focus of medical education reform. Through medical humanities courses, reflective clinical narratives, and peer support groups, educators can help students cultivate emotional connections with the physician role, facilitating the psychological transition from learning medicine to becoming a physician.

The identification of distinct latent profiles carries important implications for targeted intervention design. Students in Profile 2, characterized by moderate future work self-salience but comparatively weaker professional identity, may benefit most from structured career guidance activities that bridge their existing career aspirations with deeper professional engagement. Such activities might include shadowing experienced nurses, participating in professional conferences, or engaging in guided reflection on the meaning of nursing work. For students in Profile 3, who exhibit lower levels on both dimensions, a more comprehensive support approach is warranted. These students may require foundational interventions that simultaneously build career clarity and professional belonging. Emotional support through mentorship and counseling can address potential disengagement or career uncertainty, while carefully designed role-model interactions can provide inspirational examples that make the nursing profession more personally relevant and aspirationally accessible.

For students in the Low Future Work Self-salience–Low Professional Identity subgroup, targeted psychological counseling and meaning-centered interventions may be necessary to enhance career control beliefs and self-efficacy, thereby preventing academic disengagement and career drift. For the Moderate Future Work Self-salience–Moderate Professional Identity subgroup, identity enhancement strategies—such as phased goal-setting and social support interventions—may facilitate deeper identity integration. This approach is consistent with Branch Jr. (71) call for “professional formation” as a core educational objective and with empirical evidence demonstrating that humanities-based curricula enhance empathy, ethical reasoning, and professional identity among medical students (72). Notably, our person-centered findings suggest that such interventions may be differentially effective depending on students’ baseline profiles—a consideration that has been underexplored in prior intervention research.

From an intervention feasibility perspective, the three profiles identified by the LPA provide an empirical basis for tiered, subgroup-specific, and precision-oriented training in medical education. For the Low Future Work Self-salience–Low Professional Identity subgroup (38.5%), interventions may start with future-self narrative exercises, career-based imagery, and phased goal setting, combined with mentorship and clinical role modeling to strengthen role belongingness and value internalization, thereby reducing career uncertainty and enhancing occupational sense of mission. For the Moderate–Moderate subgroup (50.3%), which represents a transitional group with relatively high malleability, structured clinical reflection, narrative-based medical humanities activities, and peer support may facilitate consolidation of professional identity and stabilization of occupational sense of mission. For the High–High subgroup (11.2%), providing higher-challenge responsibility assignments and opportunities in research and public health practice may help maintain sustained engagement and further transform these students into resources for peer influence and role-model diffusion. Importantly, this tiered strategy is consistent with the mechanism identified in this study (Future Work Self-salience → Professional Identity → Occupational Sense of Mission).

Our finding that the High Future Work Self-salience–High Professional Identity profile exhibited the highest occupational sense of mission has important implications for identifying and nurturing future healthcare leaders. Enhancing occupational sense of mission is not only pivotal for individual student development but also essential for maintaining healthcare quality and professional ethics. Physicians with strong occupational sense of mission exhibit greater empathy, resilience, and social responsibility, which helps mitigate burnout and interpersonal conflict in clinical settings. This assertion is supported by Shanafelt, West (73) longitudinal research demonstrating that physicians with strong sense of calling and professional purpose exhibited significantly lower burnout rates and higher career satisfaction. Furthermore, West, Dyrbye (74) found that interventions enhancing professional meaning and purpose reduced emotional exhaustion among practicing physicians, suggesting that early cultivation of occupational sense of mission during medical training may have lasting protective effects. Therefore, medical educators should integrate psychological development, professional ethics, and social mission education, laying a psychological foundation for sustainable health workforce development in the post-pandemic era. At a policy level, psychological support and professional identity training should be embedded as essential components of both classroom instruction and clinical internship.

### 4.4 Limitations and future directions

Despite its theoretical and methodological contributions, this study has several limitations. First, the cross-sectional design restricts inferences regarding causal relationships and temporal dynamics among variables. Future research could adopt longitudinal or mixed-method designs to track changes in future work self-salience, professional identity, and occupational sense of mission across different stages of medical education and reveal their developmental trajectories and temporal stability. Second, the sample was drawn from three medical universities in Sichuan Province. Although representative to some extent, regional cultural norms, educational environments, and public health system differences may limit the generalizability of the findings. Future work should expand sampling to additional regions or even cross-cultural contexts to test the model’s stability and explore potential moderating effects of educational systems.

Additionally, the clinical training environment may introduce potential confounding influences. In contexts where internships involve prolonged shifts, rotating schedules, and high weekly workload, participants’ negative experiences may reflect occupational burnout or work-related distress rather than a primary psychiatric disorder. Because burnout is not classified as a mental disorder in major diagnostic systems, excluding individuals with severe psychiatric diagnoses does not necessarily rule out burnout-related influences. Such experiences may plausibly dampen future work self-salience, undermine professional identity formation, and erode occupational sense of mission, thereby affecting both the magnitude of the mediation paths and the distribution of latent profiles. Future studies should measure workload indicators and burnout using validated instruments and test burnout/workload as covariates, moderators, or alternative explanatory pathways.

In addition, although the study is framed in the post-pandemic era, we did not directly model pandemic-related exposures as focal explanatory variables to quantify whether the observed profile differences reflect a crisis-related “meaning rebound” or resilience process. Future studies could explicitly compare cohorts trained before versus after the pandemic or employ longitudinal designs to test whether pandemic-related experiences predict changes in future work self-salience, professional identity, and occupational sense of mission over time.

Third, despite the use of reverse-coded items and anonymous procedures to reduce social desirability bias, reliance on self-report questionnaires may still be subject to response style or social orientation bias. Future research should incorporate multi-source assessment methods—such as mentor evaluations, peer observations, physiological indicators, or experience sampling methodology—to enhance ecological validity. Finally, the model did not include other potentially relevant psychological variables such as career hope, psychological resilience, professional self-efficacy, or meaning-making ability. These variables may serve as chained mediators or moderators between future work self-salience and occupational sense of mission. Future studies should consider developing chained mediation models, multilevel moderation models, or even employing neuroscience-informed approaches to explore the cognitive and neural mechanisms underlying identity construction.

In summary, although this study provides new insights into medical students’ vocational psychological development in the post-pandemic era, further integration of theory, multi-context validation, and multi-method approaches will be necessary to establish a more comprehensive and dynamic theoretical framework for the cultivation of occupational sense of mission.

## 5 Conclusion

Drawing on the social and educational context of the post-pandemic era, this study systematically examined the relationships among future work self-clarity, professional identity, and occupational sense of mission in medical students, integrating variable-centered and person-centered analytical perspectives. The findings indicated that future work self-clarity not only directly predicted occupational sense of mission but also indirectly influenced it through the mediating role of professional identity. Meanwhile, the latent profile analysis revealed psychological heterogeneity within the medical student population, with distinct professional psychological types showing significant differences in occupational sense of mission levels. These results provide multi-level empirical support for understanding the motivational foundations of medical students’ professional development and offer new theoretical and practical insights for psychological interventions in medical education in the post-pandemic era.

## Data Availability

The raw data supporting the conclusions of this article will be made available by the authors, without undue reservation.
